# Dr. T. Gaillard Thomas

**Published:** 1903-04

**Authors:** 


### OBITUARY.

Dr. T. Gaillard Thomas, of New York, died suddenly of cardiac disease at Thomasville, Ga., February 28, 1903, aged 71 years. He was born on Edisto Island, S.C., near Charleston, and was graduated in medicine from Charleston Medical College in 1852. Afterward he went abroad to further pursue medical study, and then located in New York, where he practised his profession until within a short time of his death.

Dr. Thomas first cultivated the field of obstetrics and became professor of obstetrics, gynecology, and diseases of children at the College of Physicians and Surgeons. His fame as a teacher and practitioner became world-wide, and he soon limited his work to gynecology and abdominal surgery. He achieved distinction as a successor of Marion Sims at the Woman's Hospital, where as operator and teacher he divided the honors with Thomas Addis Emmet for many years.

Dr. Thomas was a writer of great force and clearness, and became known all over the world through his treatise on diseases of women, which has been translated into many tongues. The last edition was issued with the collaboration of the late Dr. Paul F. Mundé, the latter doing most of the work in revising the book. Dr. Thomas was one of the founders and became president of the American Gynecological Society.

